# Well-being and PTSD in German emergency medical services – A nationwide cross-sectional survey

**DOI:** 10.1371/journal.pone.0220154

**Published:** 2019-07-23

**Authors:** Christian Eiche, Torsten Birkholz, Eva Jobst, Christine Gall, Johannes Prottengeier

**Affiliations:** 1 Department of Anesthesiology, University Hospital Erlangen, Erlangen, Germany; 2 Faculty of Medicine, Friedrich-Alexander University Erlangen-Nuremberg, Erlangen, Germany; 3 Department of Medical Informatics, Biometry and Epidemiology, Friedrich-Alexander University Erlangen-Nuremberg, Erlangen, Germany; Technion Israel Institute of Technology, ISRAEL

## Abstract

**Background:**

Emergency medical service (EMS) can be a burdensome occupational field, and employees can be confronted with traumatizing events. Posttraumatic stress disorder (PTSD) and depression rates among paramedics are considered higher than those in the general population. In the German setting of a physician-based EMS system, the literature provides little data on PTSD or non-PTSD-related mental health or on the correlation between PTSD and well-being.

**Methods:**

The study collected data through a nationwide cross-sectional questionnaire survey of the German EMS. Next to gathering sociodemographic data, it used the 5-item World Health Organization Well-Being Index (WHO-5) and the Short Screening Scale for the DSM-IV-PTSD to assess well-being and identify indicators of depression and PTSD.

**Results:**

A total of 2,731 paramedics and emergency physicians participated in the survey; 2,684 questionnaires were submitted to analysis. The average WHO-5 score was 53.15%. A total of 43.4% of participants screened positive for possible depression, as indicated by a WHO-5 score below 50%. Female gender, older age, higher total years spent working in EMS and increased body mass index were significantly correlated with lower well-being. A total of 5.4% of respondents had a positive PTSD screening result. In particular, older employees were significantly more likely to test positive for PTSD (12.2% of those over 50 years, compared to 2.8% of those under 30 years). Positive PTSD screening results were associated with significantly lower well-being. Over an average period of 1 year, the paramedics reported perceiving a median of 2 emergency missions as mentally distressing.

**Conclusion:**

Low well-being and PTSD seem to be relevant experiences among German EMS despite their perception of low numbers of emergency responses as mentally distressing. Paramedics who have been diagnosed with PTSD should be investigated for depression and vice versa, as correlations in both directions exist. Special attention should be paid to older employees, who have significantly lower well-being and higher PTSD rates compared to younger employees.

## Introduction

Confrontation with imminent or actual death is an extreme experience. Those working in emergency medical services (EMS) may encounter serious illnesses, accidents or death in their professional career, and most paramedics have already experienced traumatizing events [1]. Furthermore, in recent years, increasing numbers of acts of aggression against EMS personnel have been reported, resulting in the passage of a law specifically for their protection in 2017 [2]. A recent study showed that 64% of EMS personnel in North-Rhine-Westphalia were victims of mission-related verbal or physical attacks within a one-year period [3].

Such mission-related acute psychosocial stress may not only cause posttraumatic stress disorder (PTSD) but may also result in depression [4,5]. There is a positive correlation between the number of fatal emergencies that paramedics have encountered and rates of PTSD and depression [6]. Even many years after a traumatic event, PTSD can persist, as recent data from the 9/11 terrorist attacks in New York show [7].

Furthermore, there has been an upsurge in EMS missions in recent years. The State of Bavaria, for example, experienced a 54% increase in emergency responses over the last ten years, while the provision of resources (i.e., ambulances) only increased by 12% over the same period [8].

Despite the knowledge that EMS can be a burdensome occupational field, the literature to date provides limited data on PTSD, especially for the German setting of a physician-based EMS system. Moreover, non-PTSD-related mental health and the correlation between PTSD and well-being in EMS have not been fully addressed [9].

A recent meta-analysis of studies outside of Germany reported the average PTSD rate among paramedics as 11% and the depression rate as 15%, with large variations among primary studies [9]. These numbers far exceed the prevalence of PTSD (2.3%) and depressive symptoms (8.1%) in the general German population [10,11].

The abovementioned findings suggest a need for more insight into PTSD and well-being among German EMS personnel, not only because of the impact that PTSD and depression have on the health system but, more importantly, because of the undisputedly large impact these disorders have on the affected individual’s health.

Information: In 2017, the German EMS employed approximately 62,000 people, of which 24% were women. [12] In Germany, the EMS is an emergency-physician-based system. In severe cases, paramedics will assist the physician in charge of the scene, while less severe cases are handled by the paramedics alone. Currently, the training of paramedics in Germany has been restructured. The former “Rettungsassistent” role required a two-year vocational training program, while the new “Notfallsanitäter” training instituted in 2014 is completed in 3 years. The “Rettungssanitäter” is trained in 13 weeks and has subordinate duties and responsibilities.

## Materials and methods

In autumn 2017, a nationwide German cross-sectional questionnaire survey was conducted to collect sociodemographic data on ambulance workers and to search for indicators of depression, PTSD and possible underlying risk factors. The study was widely advertised through information letters, posters and flyers that were sent to all German EMS stations and paramedic academies. Announcements were placed in all major German EMS journals. Relief organizations as well as labor unions and the German Association of Paramedics (Bundesverband Rettungsdienst) supported the study through their online communiques.

The survey was conducted as an online questionnaire provided via the Sosci-Survey platform. Participation was voluntary, unpaid and anonymous.

The University of Erlangen-Nuremberg’s research ethics committee approved the study beforehand in a formal decision (Decision Number 172_17B). The need for formal consent from participants was waived by the ethics committee, and voluntary participation itself served as a surrogate for consent.

To collect sociodemographic data from participants, questions regarding their age, gender, height and weight were included. Questions also addressed their professional experience, including the highest level of professional training, years’ experience working in EMS and an estimate of the total number of emergency missions handled.

The following established questionnaires were used to find indicators for depression and PTSD: The 5-item World Health Organization Well-Being Index (WHO-5) and the German version of the Short Screening Scale for DSM-IV PTSD.

The WHO-5 is used to measure subjective well-being. The questionnaire evolved from a 28-item rating scale that was initially developed to measure the quality of life of diabetes patients. The WHO-5 consists of 5 items addressing the presence of specific feelings in the last two weeks. The items “I have felt cheerful and in good spirits”, “I have felt calm and relaxed”, “I have felt active and vigorous”, “I woke up feeling fresh and rested” and “My daily life has been filled with things that interest me” are rated on a 6-point scale from 0 (never) to 5 (all the time). The subscales are summed to produce a score ranging from 0 to 25, which is multiplied by 4 to obtain a percentage scale value from 0% (worst well-being) to 100% (best well-being). The WHO-5 Index avoids symptom-related language and measures well-being in terms of positive subjective quality of life. Scores of less than 50% are indicative of depression. A value under 28% indicates possible major depression. A score difference of more than 10% was defined as the threshold for clinical relevance, in accordance with previous literature [13].

The German version of the Short Screening Scale for DSM-IV-PTSD was used to investigate the potential presence of PTSD. The questionnaire contains seven items, five from the *avoidance* symptom cluster and two from the *arousal* symptom cluster. An item is considered positive if the symptom occurs at least 2–4 times a week. All positive items are summed to produce a total score ranging from 0 to 7 points. For a positive PTSD screening diagnosis, a minimum of 4 points is required, meaning four or more symptoms are present at least 2–4 times a week [14,15].

The participants were asked to estimate subjectively the number of mentally distressing or traumatic emergency missions they had undertaken in their entire career and in the last 12 months. To estimate the long-term frequency of mentally distressing or traumatic emergency runs, the number of such missions was divided by the estimated number of total emergency responses in each paramedic’s professional life. In addition, the participants were asked whether, during their most traumatic incidents, they felt helpless or were very afraid or horrified.

### Statistical analysis

Data analysis was conducted in R version 3.4.3 [16]. General characteristics are presented as medians or means with standard deviations or 95% confidence intervals where applicable.

Risk factors for possible depression (WHO-5 < 50%) and PTSD (positive PTSD items > = 4) were analyzed by multivariate logistic regression with p-values from ANOVA type III tests for categorical predictor variables.

Originally, the questionnaire aimed to describe both physicians and paramedics in an equally balanced manner. However, only 2.9% of the participants in the survey were emergency physicians. These participating physicians were included in the final statistical analysis to avoid result-dependent data evaluations. A separate regression model that excluded the small number of physicians was also calculated, and it identified the same risk factors as the main regression model. As the physician cohort was small in number and did not influence the findings, we excluded this group from the narrative and only discussed the results in terms of paramedics, even though the data gathered from physicians remained part of the data set.

## Results

A total of 2,731 paramedics and emergency physicians completed the questionnaire; 2,684 of these questionnaires fulfilled the quality criteria (less than 20% missing answers to all items) and were submitted to analysis.

Table 1 presents an overview of basic sociodemographic characteristics of the participants and presents the distribution of the mean WHO-5 scores and PTSD. The male-to-female ratio corresponds approximately to the actual distribution in the German EMS [12].

**Table 1:** Sociodemographic characteristics and distribution of WHO-5 scores and PTSD.

		WHO-5 score			PTSD			
		Mean	Standard deviation	Positive depression screening*	No		Yes	
					N	(%)	N	(%)
Age, years	18–30 (N = 1167)	56.11	18.67	36.7%	1134	97.2%	33	2.8%
	31–40 (N = 758)	53.30	19.38	43.5%	721	95.1%	37	4.9%
	41–50 (N = 525)	48.23	20.81	54.9%	479	91.2%	46	8.8%
	51–70 (N = 230)	49.08	21.72	50.9%	202	87.8%	28	12.2%
Gender	Male (N = 2140)	53.37	20.04	42.8%	2013	94.1%	127	5.9%
	Female (N = 540)	52.52	18.80	45.7%	523	96.9%	17	3.1%
Weight	Underweight (N = 17)	51.29	23.29	52.9%	15	88.2%	2	11.8%
	Normal weight (N = 1061)	55.80	19.62	39.2%	1009	95.1%	52	4.9%
	Pre-obese (N = 1013)	52.71	19.53	43.3%	960	94.8%	53	5.2%
	Obese (N = 586)	49.26	19.89	50.9%	548	93.5%	38	6.5%
Highest level of professional training	Other qualification (N = 19)	61.05	16.16	21.1%	18	94.7%	1	5.3%
	Notfallsanitäter in training (N = 98)	64.24	18.07	22.4%	96	98.0%	2	2.0%
	Rettungssanitäter (N = 483)	55.60	19.41	38.9%	458	94.8%	25	5.2%
	Rettungsassistent (N = 1177)	50.63	19.66	48.5%	1101	93.5%	76	6.5%
	Notfallsanitäter (N = 832)	52.99	20.05	43.3%	792	95.2%	40	4.8%
	Emergency physician (N = 75)	62.19	15.75	28.0%	74	98.7%	1	1.3%
Years of service	0–5 (N = 928)	57.06	18.70	34.4%	903	97.3%	25	2.7%
	6–15 (N = 883)	52.61	19.25	45.0%	840	95.1%	43	4.9%
	>15 (N = 866)	49.60	20.83	51.6%	791	91.3%	75	8.7%

* pos. depression screening indicated by a WHO-5 score <50%

### 5-item World Health Organization Well-Being Index

The mean WHO-5 score among the paramedics was 53.15% (SD 19.82). In men, the average score was 53.37%, while in women, it was 52.52%. Positive depression screening, as indicated by a score of less than 50%, was found in 43.4% of the participants; scores of less than 28%, which were a possible indicator of major depression, were identified in 15.1%. The odds ratio of having a score under 50% was 1.59 (CI: 1.29–1.97) for women compared to men.

It was possible to identify various factors that had a negative influence on a person’s well-being. Employees with more than 15 years of employment had an average WHO score of 49.6%. Of these veteran paramedics, 51.6% had scores of less than 50%; additionally, the odds ratio of a score less than 50% compared to paramedics with 0–5 years of work experience was 1.56 (CI: 1.11–2.21).

A higher number of emergency missions per year handled by the individual was significantly correlated with lower well-being. Age, gender, body mass index (BMI), level of paramedic training and total working years also had a significant influence on well-being (Table 2).

**Table 2:** Risk factors for a well-being score of <50%.

Parameter		OR	95%-CI <	95%-CI>	p
Age	18–30 years	1			0.0039
	31–40 years	1.1707	0.9214	1.4872	
	41–50 years	1.8275	1.3067	2.5594	
	>50 years	1.5093	0.9975	2.2860	
Gender	Male	1			< 0.001
	Female	1.5922	1.2853	1.9733	
Weight	Underweight	2.4103	0.8381	7.4085	0.0035
	Normal weight	1			
	Pre-obese	1.1083	0.9182	1.3378	
	Obese	1.4508	1.1662	1.8055	
Highest level of professional training (years of training)	Notfallsanitäter trainee	0.5152	0.2989	0.8558	< 0.001
	Rettungssanitäter (13 weeks)	0.9071	0.7137	1.1520	
	Rettungsassistent (2 years)	1			
	Notfallsanitäter (3 years)	0.6810	0.5605	0.8267	
	Emergency physician (university)	0.3235	0.1807	0.5573	
Years of service	0–5	1			0.0057
	6–15	1.4569	1.1491	1.8485	
	>15	1.5622	1.1074	2.2050	
Missions per year	(steady increment)	1.0003	1.0001	1.0005	0.0018

**Fig 1:**
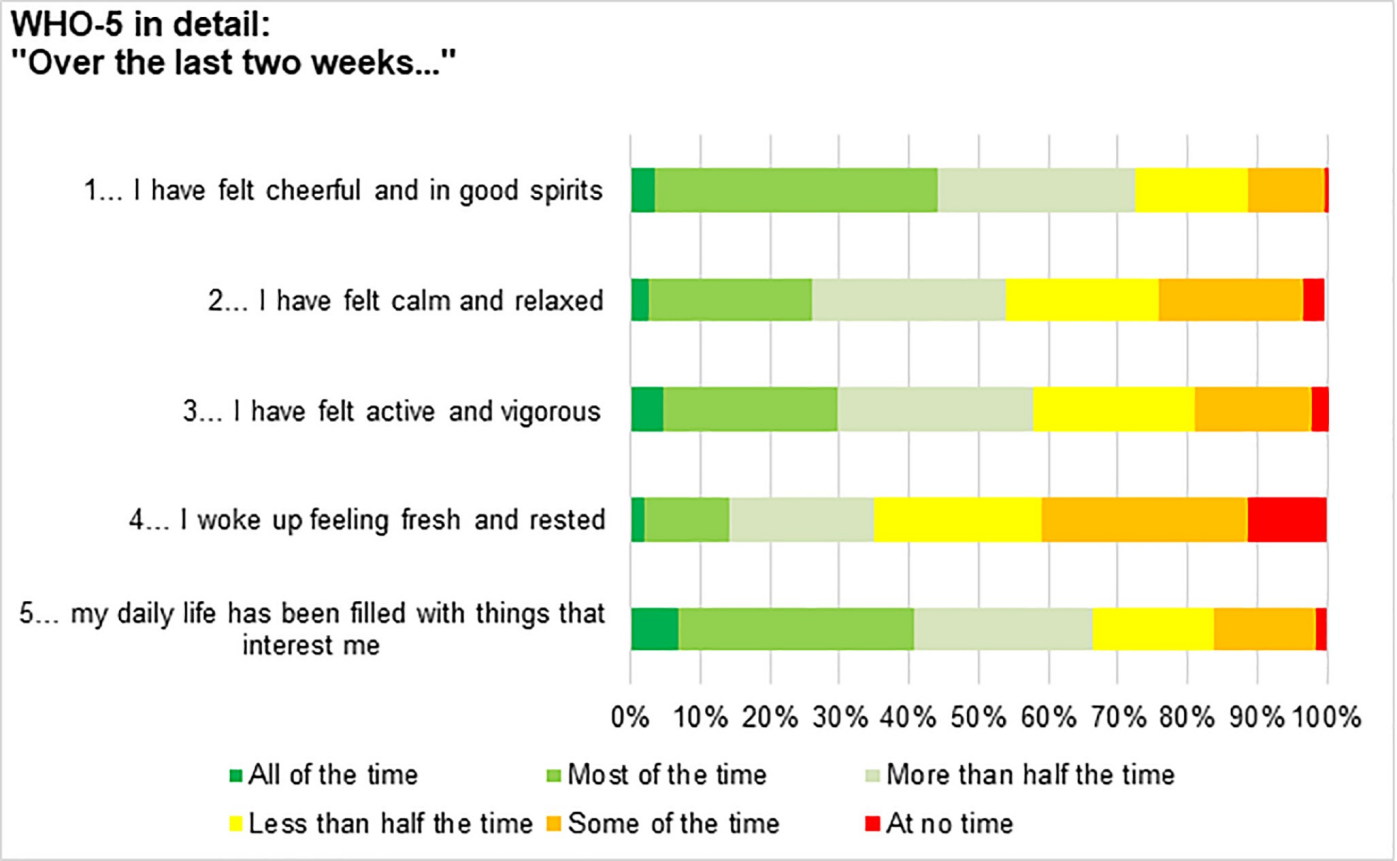
Detailed answers of the WHO-5 questionnaire.

Additional single item analysis showed that the question "… I woke up feeling fresh and rested" showed particularly negative results. In total, 65% of participants woke up fresh and rested less than half of the time, while 11.3% never woke up feeling fresh and rested. Fig 1 presents a detailed overview of the answers to the WHO-5 questionnaire.

### PTSD screening

A positive PTSD screening was found in 5.4% of paramedics. A total of 28.3% of participants had experienced a situation during their EMS work during which they felt very afraid or horrified, and 39.3% of participants had encountered a situation in which they experienced helplessness.

A total of 3.1% of the women were screened positive for PTSD, compared to 5.9% of men. The odds ratio for women to have a positive PTSD screening was 0.62 compared to men, but this difference did not meet the criteria for statistical significance (CI: 0.32–1.11; p = 0.113).

Older employees in particular had a significantly higher rate of positive PTSD screenings: 12.2% for those over 50 years of age compared to 2.8% for those under 30; OR (over 50 years vs. under 30 years) 3.75 (CI 1.64–8.55).

Among the participants with more than 15 years of service, 8.7% had a positive PTSD screening, compared to 2.7% for personnel with less than 5 years on the job. However, this difference was not statistically significant (p = 0.145).

Table 3 shows the risk factors for PTSD.

**Table 3:** Risk factors for a positive PTSD screening.

Parameter		OR	95%-CI <	95%-CI>	p
Age	18–30 years	1			0.011
	31–40 years	1.5462	0.8494	2.8334	
	41–50 years	2.8124	1.3517	5.8281	
	>50 years	3.7534	1.6436	8.5480	
Gender	Male	1			0.113
	Female	0.6218	0.3215	1.1128	
Weight	Underweight	2.7018	0.1418	15.4790	0.436
	Normal weight	1			
	Pre-obese	0.7552	0.4938	1.1536	
	Obese	0.9522	0.6005	1.4988	
Highest level of professional training (years of training)	Rettungsassistent (2 years)	1			0.0057
	Notfallsanitäter trainee	0.9726	0.1507	3.5503	
	Rettungssanitäter (13 weeks)	1.3518	0.7834	2.2673	
	Notfallsanitäter (3 years)	0.5970	0.3893	0.9036	
	Physician (university)	0.1471	0.0082	0.6989	
Years of service	0–5	1			0.145
	6–15	1.8386	0.9752	3.5321	
	>15	2.0200	0.9065	4.6184	
Missions per year	Steady	1.0001	0.9996	1.0005	0.753

**Fig 2:**
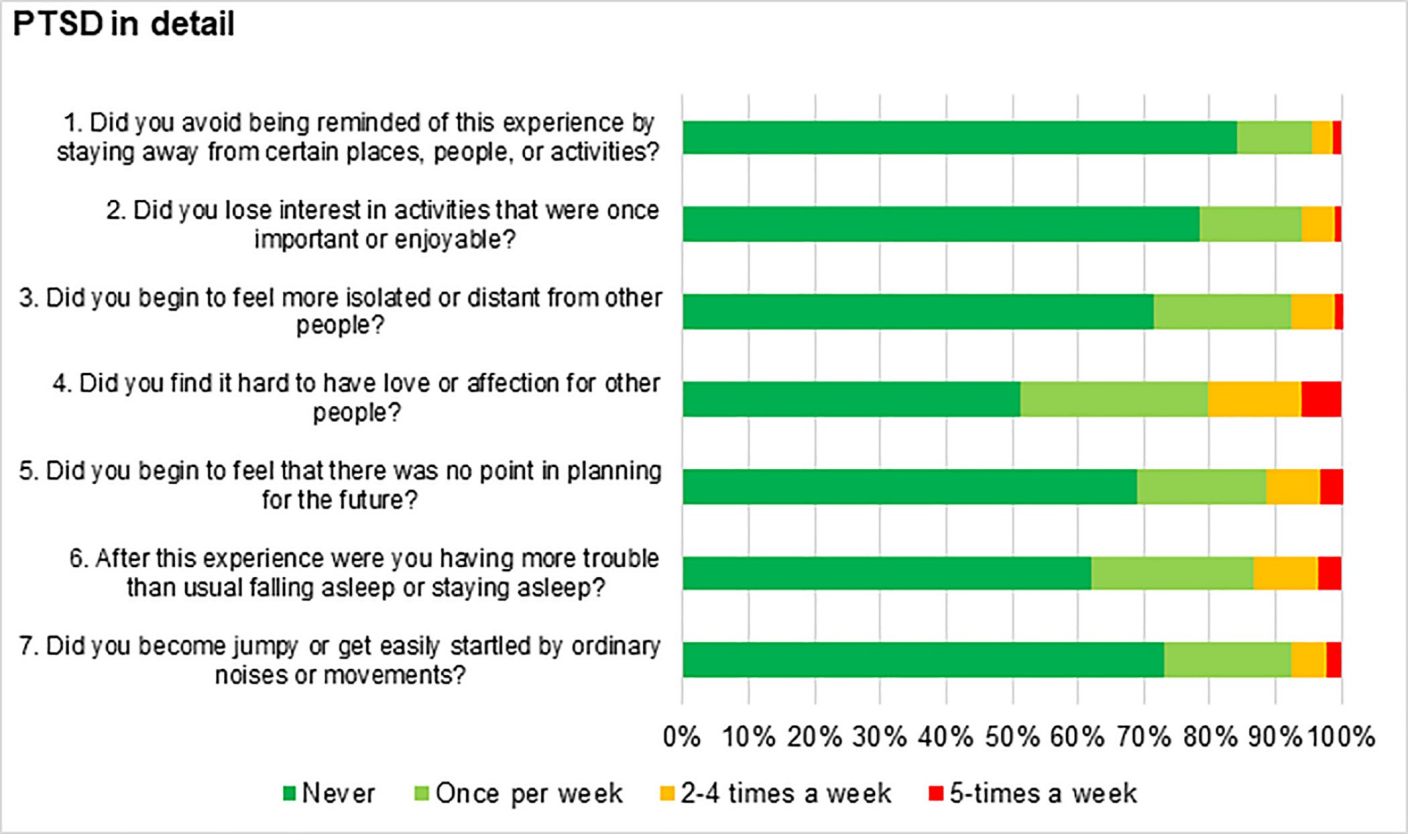
Detailed answers to the PTSD screening questionnaire.

The single item analysis showed that more than 20% of participants stated that they found it hard to have love or affection for other people at least half of the time, but only 4.4% showed avoidance of certain places, people or activities. Fig 2 presents the detailed answers gathered from the PTSD questionnaire.

A mean of 1 out of 266 missions was perceived as significantly mentally distressing, with a wide range among individuals. In men, it was 1 in 300, while for women, it was 1 in 200 emergency missions. The paramedics self-evaluated emergency missions as distressing according to their own measure.

**Fig 3:**
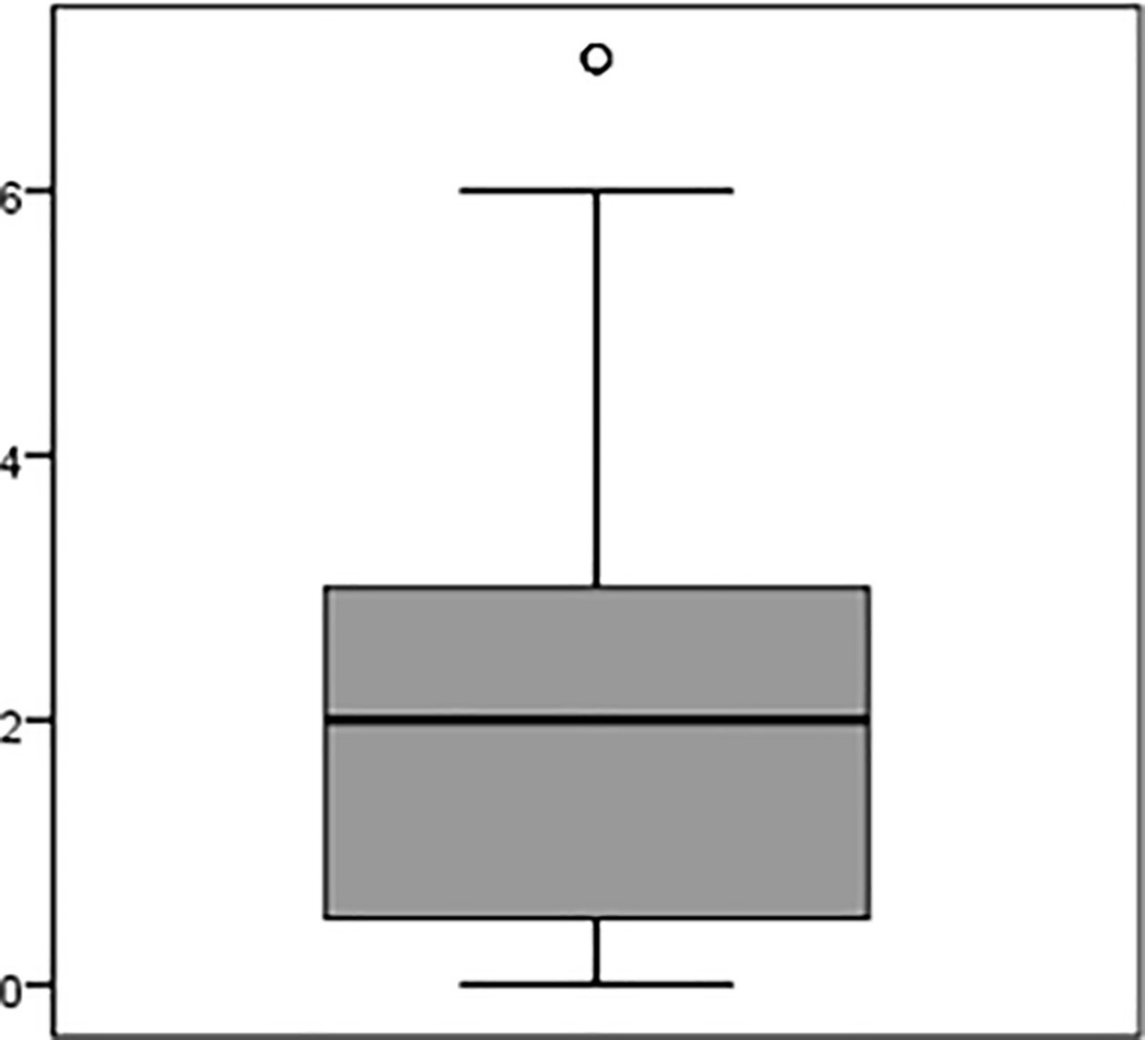
Number of particular mentally distressing emergency responses in the last 12 months.

Fig 3 shows that in the 12 months prior to the study, the participants had a median of two emergency missions that they perceived as particularly mentally distressing or traumatic, and only a few individuals had experienced more than 6 such incidents.

### Relationship between PTSD screening and the WHO-5 well-being score

Participants with a particularly low WHO-5 score (<28%) showed a positive PTSD screening in 19.5% of cases. PTSD also had additional negative implications for the participants’ well-being, meaning that the more symptoms of PTSD that were present, the lower the individual’s well-being was.

**Fig 4:**
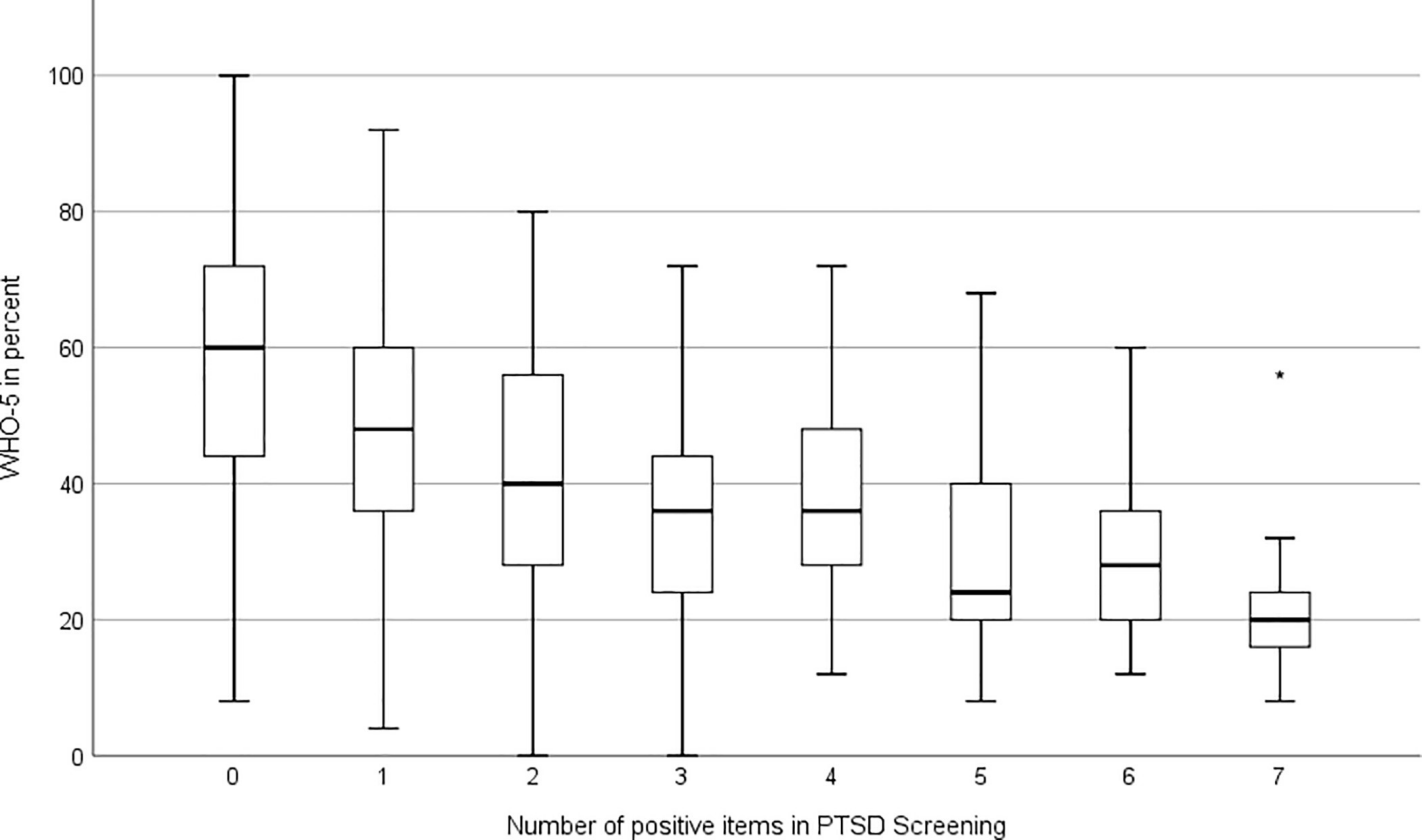
Correlation between the WHO-5 score and the number of positive items on the PTSD screening.

**Fig 5:**
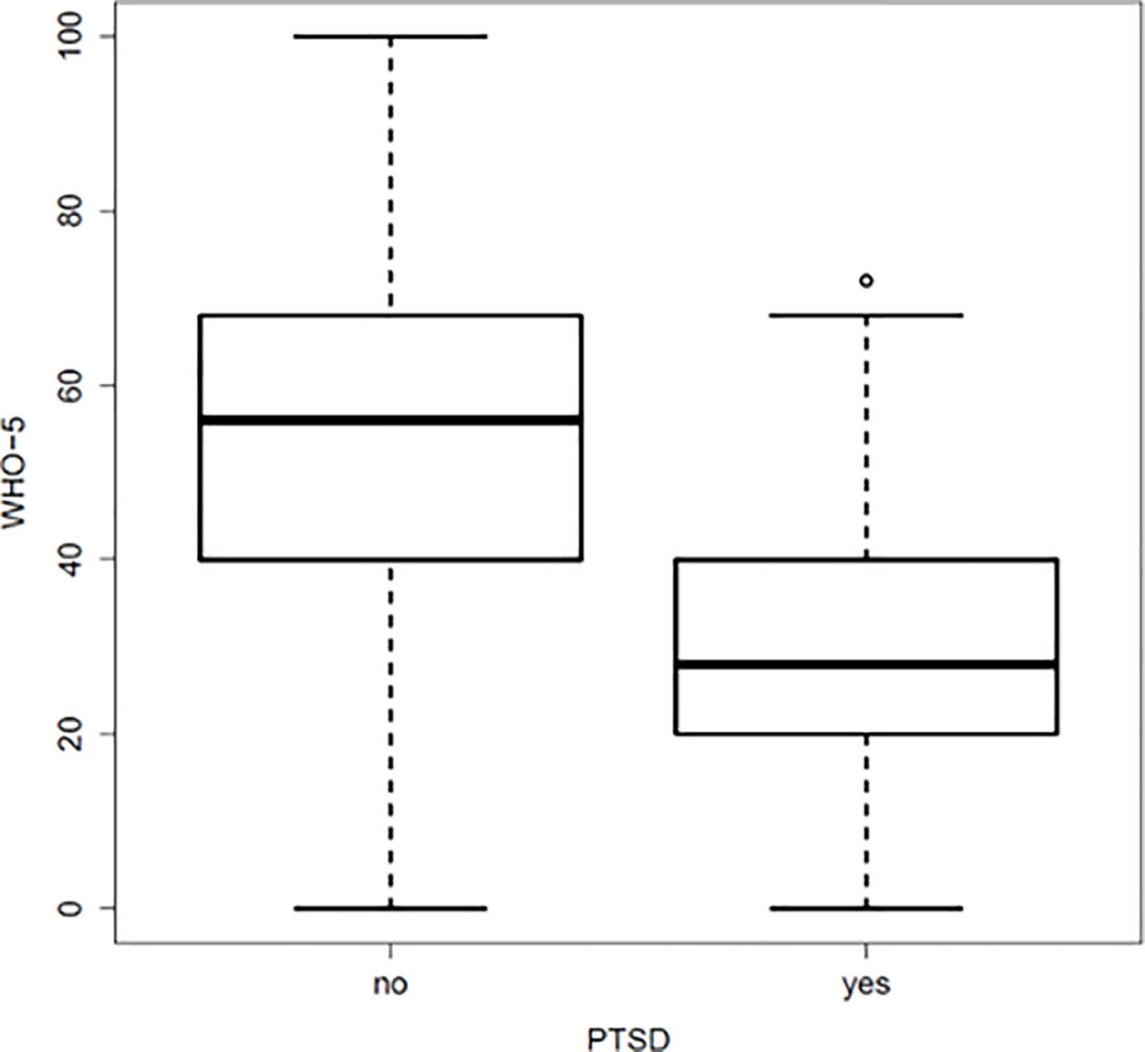
Correlation between positive PTSD screening results and the WHO-5 score.

For the two disease entities, the point-biserial correlation was calculated at -0.258 with a 95% confidence interval of [-0.293; -0.223] (Figs 4 and 5).

## Discussion

Mental health disorders have a large impact on the individual patient and on entire health systems and economies. They are the second most frequent reason for the inability to work in Germany. The number of days off work due to mental disorders has increased threefold within the last 20 years [17,18]. Although prehospital emergency medicine has been identified as an occupation with a high number of risk factors for mental health disorders, limited data are available regarding PTSD and well-being against the background of Germany’s EMS.

A recent systematic review and meta-analysis of EMS personnel worldwide showed a multinational PTSD rate of 11% and a depression rate of 15%. The authors emphasized that at the time of their analysis, there were very limited data available regarding non-PTSD-related mental health. They concluded that ambulance personnel remained an underresearched population [9].

In the present study, 5.4% of paramedics had a positive PTSD screening, compared to the 2.3% of the general population of Germany diagnosed with PTSD.

Compared to studies from other countries worldwide, a relatively low rate of positive PTSD screening tests in the German cohort of paramedics has been reported. The reasons for this hopeful finding remain speculative, but it is conceivable that the overall conditions within the German EMS system play an important role. Generous funding and the geographic properties of this densely populated, high-income country allow for close coverage of EMS resources. The availability of emergency physicians, a relatively low deployment threshold, short response times, short transfer distances and a high level of training among paramedics presumably contribute to the relief of an otherwise heavy burden. These assumptions are supported by Streb et al., who described a PTSD rate of 4.3% in German-speaking Switzerland, where the conditions for paramedics are comparable to those in Germany [19]. Similar findings were made in Hawaii, where Mishra et al. reported a PTSD rate of 4% [20]. Alarmingly high PTSD rates were recently reported from Poland. In a cohort of 101 Polish paramedics, 40% were identified with potential PTSD [21]. Another study from Poland reported 34.8% of 270 paramedics with a preliminary diagnosis of PTSD but also showed factors of posttraumatic growth like greater appreciation of life [22]. The reasons for this exceptionally high rates remain unclearIn addition to this general geographical and economic framework, German EMS includes a specially trained crisis intervention team that offers psychological support for patients, relatives, bystanders and paramedics after traumatizing events. Furthermore, most EMS stations have specially trained employees who serve as confidants for other paramedics. As a result of both these features, paramedics in Germany have easy access to psychological help if they are involved in traumatizing events.

When the participants were asked “How many emergency responses did you perceive as mentally distressing or traumatic in the last twelve months?”, they reported perceiving a median of 2 emergency responses per year as traumatic or mentally distressing. This seems to be a relatively low rate considering that ambulance workers come in contact with critically ill or injured patients on a daily basis. In comparison, paramedics from Israel reported five particularly grueling incidents in a period of only six months. [23]

A total of 12.2% employees over 50 years of age had positive PTSD screenings, almost four times the prevalence for employees younger than 30 years. There may be a cumulative aspect of the development of PTSD after traumatization, and preventive measures should place extra emphasis on older employees. However, these findings are in contrast to a recent study from Pakistan, where younger EMS personnel was more likely to have increased severity of symptoms of PTSD. [24]

Compared to the average German population, which has a mean well-being of 64.7% [25], the overall well-being level among paramedics was considerably lower at 53.15%. Differences of 10% or more are considered clinically relevant and usually serve as the threshold for the step-up of therapeutic measures. Compared to other EU countries, life satisfaction among rescue workers is similar to that of the population of the country with the worst well-being in Europe, which is 51.8%. [25]

A positive depression screening result was found for 43.4% of the participants. This is a much higher rate than in former studies conducted outside of Germany, which reported an average depression rate of 15% is described [9]. This 15% marker corresponds with the number of participants in our study who had scores of less than 28% and thus have potential major depression.

The present study found that the participants had particularly low scores for the question “I woke up feeling fresh and rested” on the WHO-5 questionnaire, a finding that has a large impact on general well-being. It is beyond the scope of this investigation to present the causes for this finding, but future research into this particular aspect of well-being is strongly encouraged.

Consistent with higher positive PTSD screening rates, older employees also scored lower for well-being. The average well-being score for individuals over the age of 50 years was only 49.08%, compared to 56.11% in those under 30. The odds ratio for possible depression was 1.50, which was significant. Next, obesity, female gender and long-term service were identified as factors significantly associated with poor well-being. It could also be shown that the number of missions per year had a significant influence on well-being.

A strong correlation between possible PTSD and decreased well-being was found. Participants with a well-being index below 28% had an almost 4-fold rate of possible PTSD. Therefore, when a paramedic presents with clinical depression, PTSD must be considered and tested for. Complementary to that, positive PTSD screenings showed strong correlations with poor well-being. However, the data could not elucidate whether a given stress disorder directly caused low well-being or whether the decrease in well-being could be explained indirectly through the increased risk of depression, which has that impact by itself.

### Strengths of the study

The study provides a large data set addressing PTSD and well-being in those working in EMS. The cohort includes employees with little work experience as well as veteran colleagues and covers all levels of training. We are confident that a study population representative of the entire EMS can be provided. Because the request to participate was sent through various media outlets and with the support of professional associations, labor unions and employers alike, employees from all aspects of German EMS could be reached.

### Limitations of the study

Naturally, there were some limitations to our study. Most importantly, selection bias cannot be ruled out in voluntary and anonymous questionnaires. It cannot be guaranteed that the sample was fully representative of the entire workforce of German paramedics. Putting it more openly, it cannot be discerned whether healthy individuals felt more motivated to participate or whether those actually affected by depression and PTSD felt more compelled to do so. Furthermore, the “healthy-worker effect” may have led to a generally better health status within the study population compared to the actual target population because those who left EMS for health reasons could no longer be characterized [26].

Finally, the screening questionnaires that were used were designed to produce clinical suspicion. The definitive diagnosis of PTSD or depression is still based exclusively on a full psychiatric exploration.

## Conclusion

Low levels of well-being and possible PTSD among paramedics present significant challenges to the German EMS. Although PTSD may occur less frequently in Germany than in other countries, there is a need for action to avert personal harm to paramedics and to promote root-cause analyses regarding occupational and cohort aspects. Special attention must be paid to the existing correlation between well-being and PTSD. If a paramedic is identified as suffering from PTSD, depression should be investigated, and vice versa.

It is conceivable that at-risk groups (especially paramedics with a long employment record) should undergo regular screenings for depression and PTSD. Resilience training and counselling could be offered to those exposed to trauma and other stressors. Unfortunately, it is not possible to make any specific recommendations based on the current data.

Finally, the data may serve as a source of hypotheses for future follow-up studies. Changes in well-being and PTSD scores may reflect the effectiveness of implemented measures and could serve as a warning for unwanted developments in the profession of EMS, especially in the current context of an accelerating skilled labor shortage in Germany’s health system.

## Supporting information

S1 Text Questionnaire in German. (PDF)

S2 Text Questionnaire in English. (PDF)
