# Supplementary material for: Well-being and PTSD in German emergency medical services – A nationwide cross-sectional survey
Source: PLoS One. 2019 Jul 23;14(7):e0220154. doi: 10.1371/journal.pone.0220154 (PMC6650072; doi:10.1371/journal.pone.0220154)
Supplement: S1 Text — (PDF) [file pone.0220154.s001.pdf]

1. Nennen Sie uns bitte Ihr Alter in ganzen Jahren

2. Nennen Sie bitte Ihr Geschlecht

- Männlich
- Weiblich

3. Bitte nennen Sie uns Größe und Gewicht

- Körpergröße in cm
- Gewicht in kg

4. Ihr Ausbildungsstand:

- Notfallsanitäter
- Notfallsanitäter in Ausbildung
- Rettungsassistent
- RAiP
- Rettungssanitäter
- sonstige Rettungsdienst-Qualifikation
- Krankenpfleger
- Arzt / Notarzt

5. Über welche Berufserfahrung (im Rettungsdienst) verfügen Sie? In ganzen Jahren, abgerundet.

Wer unter 1 Jahr dabei ist, schreibt bitte 0.

- Anzahl Berufsjahre

6. Wie viele Einsätze in der Notfallrettung haben Sie in Ihrem Berufsleben bisher absolviert?

Eine sinnvolle Schätzung reicht!

- Anzahl Einsätze

7. WHO-5-Fragebogen zum Wohlbefinden:

Die folgenden Aussagen betreffen Ihr Wohlbefinden in den letzten 2 Wochen. Bitte markieren Sie bei jeder Aussage die Rubrik, die Ihrer Meinung nach am besten beschreibt, wie Sie sich in den letzten 2 Wochen gefühlt haben.

In den letzten 2 Wochen…	Die ganze Zeit	Die meiste Zeit	Mehr als die Hälfte der Zeit	Weniger als die Hälfte der Zeit	Manchmal	Zu keinem Zeitpunkt
… war ich froh und guter Laune						
… habe ich mich ruhig und entspannt gefühlt						
… habe ich mich aktiv und lebendig gefühlt						
… bin ich froh und erholt aufgewacht						
… war mein tägliches Leben mit Dingen gefüllt, die mich interessieren						

8. Haben Sie schon schwerwiegende, psychisch belastende oder gar traumatisierende Einsätze erlebt?

- Anzahl derartiger Einsätze insgesamt
- Anzahl derartiger Einsätze in den letzten 12 Monaten
- Anzahl derartiger Einsätze in den letzten 30 Tagen

9. Während des schlimmsten Erlebnisses...

- fühlten Sie sich hilflos? (ja/nein)
- hatten Sie starke Angst oder waren voller Entsetzen? (ja/nein)

10. Short Screening Scale for DSM-IV Post-traumatic Stress Disorder

Im letzten Monat	Überhaupt nicht	Einmal pro Woche oder seltener / manchmal	2-4mal pro Woche / die Hälfte der Zeit	5-mal pro Woche / fast immer
Haben Sie sich bemüht, Aktivitäten, Menschen oder Orte zu vermeiden, die Sie an das Erlebnis erinnern?				
Haben Sie deutlich weniger Interesse an Aktivitäten, die vor dem Erlebnis wichtig für Sie waren, oder haben Sie sie deutlich seltener unternommen?				
Fühlten Sie sich den Menschen Ihrer Umgebung deswegen entfremdet und anders?				
Fühlten Sie sich abgestumpft oder taub (z. B. nicht weinen können oder sich unfähig fühlen, liebevolle Gefühle zu erleben)?				
Hatten Sie das Gefühl durch das Erlebnis, dass sich Ihre Zukunftspläne und Hoffnungen nicht erfüllen werden (z. B. dass Sie keine Kinder haben oder dass Sie keinen Erfolg im Beruf haben würden)?				
Hatten Sie Ein- oder Durchschlafstörungen seit dem Erlebnis (d.h. vorher noch nicht)?				
Waren Sie nervös oder schreckhaft (z. B. wenn jemand hinter Ihnen Geräusche macht)?				
