# Supplementary material for: Well-being and PTSD in German emergency medical services – A nationwide cross-sectional survey
Source: PLoS One. 2019 Jul 23;14(7):e0220154. doi: 10.1371/journal.pone.0220154 (PMC6650072; doi:10.1371/journal.pone.0220154)
Supplement: S2 Text — (PDF) [file pone.0220154.s002.pdf]

1. Please tell us your age in whole years

2. Please tell us your gender

- male
- female

3. Please tell us your height and weight

- height in cm
- body weight in kg

4. Your highest level of professional training:

- Notfallsanitäter
- Notfallsanitäter in Ausbildung
- Rettungsassistent
- RAiP
- Rettungssanitäter
- other qualification
- nurse
- emergency physician

5. How long have you been working in EMS? (In whole years)

If you have been working shorter than a year, please enter „0“

- years of service

6. How many emergency missions have you handled in your career?

An estimate number is sufficient!

- Number of emergency missions

7. WHO (Five) Well-Being Index

Please indicate for each of the five statements which is closest to how you have been feeling over the last two weeks.

Over the last two weeks…	All of the time	Most of the time	More than half of the time	Less than half of the time	Some of the time	At no time
I have felt cheerful and in good spirits						
I have felt calm and relaxed						
I have felt active and vigorous						
I woke up feeling fresh and rested						
My daily life has been filled with things that interest me						

<u>8. Did you ever handle mentally distressing or traumatic emergency missions?</u>

- Number of mentally distressing missions in your entire career
- Number of mentally distressing missions in the last 12 months

<u>9. During your most traumatic incident…</u>

- … did you feel helpless? (yes/no)
- … have you been very afraid or horrified? (yes/no)

10. Short Screening Scale for DSM-IV Post-traumatic Stress Disorder

In the past month…	At no time	Once per week or less	2-4 times a week	5-times a week / all of the time
Did you avoid being reminded of this experience by staying away from certain places, people, or activities?				
Did you lose interest in activities that were once important or enjoyable?				
Did you begin to feel more isolated or distant from other people?				
Did you find it hard to have love or affection for other people?				
Did you begin to feel that there was no point in planning for the future?				
After this experience were you having more trouble than usual falling asleep or staying asleep?				
Did you become jumpy or get easily startled by ordinary noises or movements?				
